# Unlocking the Power of Gesture: Using Movement-Based Instruction to Improve First Grade Children’s Spatial Unit Misconceptions

**DOI:** 10.3390/jintelligence11100200

**Published:** 2023-10-13

**Authors:** Eliza L. Congdon, Susan C. Levine

**Affiliations:** 1 Department of Psychology, Williams College, Williamstown, MA 01267, USA; 2 Department of Psychology, The University of Chicago, Chicago, IL 60637, USA

**Keywords:** gesture, spatial thinking, units, linear measurement, desirable difficulty

## Abstract

Gestures are hand movements that are produced simultaneously with spoken language and can supplement it by representing semantic information, emphasizing important points, or showing spatial locations and relations. Gestures’ specific features make them a promising tool to improve spatial thinking. Yet, there is recent work showing that not all learners benefit equally from gesture instruction and that this may be driven, in part, by children’s difficulty understanding what an instructor’s gesture is intended to represent. The current study directly compares instruction with gestures to instruction with plastic unit chips (Action) in a linear measurement learning paradigm aimed at teaching children the concept of spatial units. Some children performed only one type of movement, and some children performed both: Action-then-Gesture [AG] or Gesture-then-Action [GA]. Children learned most from the Gesture-then-Action [GA] and Action only [A] training conditions. After controlling for initial differences in learning, the gesture-then-action condition outperformed all three other training conditions on a transfer task. While gesture is cognitively challenging for some learners, that challenge may be desirable—immediately following gesture with a concrete representation to clarify that gesture’s meaning is an especially effective way to unlock the power of this spatial tool and lead to deep, generalizable learning.

## 1. Introduction

Gestures are movements made by the hands that are typically produced simultaneously with spoken language and can supplement that spoken language by pointing out referents in the environment, representing semantic information, emphasizing important points, or showing spatial relations (see McNeill 1992 for a classification system). Their specific features and ability to link language with sensorimotor or spatial representations make gestures a promising tool to improve spatial thinking in science, technology, engineering, and math (STEM) disciplines (e.g., Weisberg and Newcombe 2017). Indeed, intentionally incorporating representational hand gestures into instruction improves learning outcomes in content areas as diverse as pre-algebraic equation balancing (e.g., Cook et al. 2013; Goldin-Meadow 2011); understanding geological processes in geosciences (Atit et al. 2015); learning stereoisomers in organic chemistry (Ping et al. 2022); and more general spatial skills like mental rotation (Levine et al. 2018; Ping et al. 2011; Wakefield et al. 2019). Yet, there is recent work showing that not all children benefit equally from gesture instruction (e.g., Congdon et al. 2018). To better understand both the power of gestures and their potential shortcomings as a tool to improve spatial thinking, the current study compares gestures to a similar but distinct category of movement—actions-on-objects—to measure their differential impact on learning, and test whether learning outcomes might be improved beyond the benefit of action and gesture alone by teaching both types of representation (action and gesture) in the same instructional setting.

While gestures are certainly a type of action, in pedagogical or demonstrative contexts, they differ from other kinds of action-on-objects in a few key ways. First, they synchronize more tightly with spoken language than action-on-objects, even in contexts where both actions and gestures are being used to demonstrate the use of the same objects (Church et al. 2014; Wakefield et al. 2023). Secondly, gestures do not involve the direct use or manipulation of objects, which has two clear implications. The first is that without needing to manipulate objects, gestures can be more flexible in their form. They require less veridical handshapes and less precise motor planning than would be required by interaction with an object. This means that gestures can selectively represent the important features of an object in a given context. For example, a speaker might choose to use gesture to represent the length or movement trajectory of an object while ignoring an irrelevant feature like the object’s texture or color (see Uttal et al. 2009 for a review of the ways in which irrelevant features of objects can lead to worse learning outcomes). The second implication of the fact that gestures do not directly interact with objects is that gestures have no lasting end state. When a gesture is complete, there is no concrete trace of the movement for a speaker or a listener to revisit. 

These subtle differences in features between gestures and actions-on-objects can lead to dramatically different conceptualizations of these movement types in the mind of a learner, and may explain why gestures can lead to longer-lasting, more flexible learning than actions-on-objects (see Congdon and Goldin-Meadow 2021 for a review). At the same time, gestures may be less accessible than actions on objects to learners without adequate cognitive resources (Congdon 2022) or the prior content knowledge that enables them to understand the referents of the gestures (Congdon et al. 2018). Indeed, toddlers can understand the goal of an action or failed action before they can understand the meaning of a perceptually similar iconic gesture (Novack et al. 2015), and children and even adults have difficulty interpreting gestures as meaningful hand movements if they are not given enough context in which to interpret them (Wakefield et al. 2018; Novack et al. 2016). So, on one hand, gestures may be a powerful tool for spatial learning because they can represent actions and ideas in a flexible, generalizable way and because they can link abstract representations to spoken language, but on the other hand, that power is not useful for those who do not understand the gestures.

If we agree that gesture can be a useful spatial tool to improve thinking and reasoning in spatial contexts, there is value in determining how to help children access the tool during instruction, even if they may not be able to spontaneously deduce the representational meaning of the gesture on their own. One way to successfully incorporate gesture into instruction for these learners might be to pair it, within the same lesson, with a more concrete, action-based representation. In other words, providing children with two representations of the same concept might mean that children are able to extract the benefits of both types of representation (action and gesture) and, at the same time, use the analogical similarities between the two to help clarify the gesture’s meaning. This hypothesis stems from the principles of category learning and verb learning in the domain of language. Multiple exemplar training (MET) shows that giving children more than one exemplar of a category helps them extract the relevant characteristics of that category, remember the rules better, and apply them to novel situations (e.g., Smiley and Huttenlocher 1995; Gentner 2003; Horst et al. 2013). In the domain of mathematics, there is evidence that providing young children with varied examples of perceptually distinct triangles can better help them to extract the defining features of triangles (Smith et al. 2014). Moreover, it has been established for some time that variability in instruction is generally more powerful than uniformity (Bruner 1966). Under this logic, keeping instruction time and quantity constant, we would predict that providing both a plastic unit chip and a gesture as representations of a unit within one lesson should be more effective at improving children’s learning outcomes than providing either representation alone. 

On the other hand, it is possible that children will do particularly poorly when given multiple representations, especially if they are unable to make the appropriate conceptual links between the two movement types, or if they become overwhelmed by too much information in a short lesson. For example, in order to extract the benefits of multiple exemplars, it could be necessary to present action and gesture simultaneously, or to interweave them, especially given recent evidence that children learn more effectively from instructor gesture if they have higher working memory resources (Congdon 2022). Relatedly, children may do best when only presented with one representation that they can focus on mastering. For example, evidence from language learning shows that infants learning a new verb on a more complex task learned and extended that meaning best when only exposed to one exemplar as compared to four (Maguire et al. 2008), and infants learning spatial prepositions learned best when shown the same exemplar multiple times, rather than different exemplars (Casasola 2005). A recent review argues that when learning more abstract components of language, like verbs, children benefit more from multiple exemplars at later points in their learning trajectory (Imai and Childers 2020). Each of these studies suggests a ‘less is more’ hypothesis for naïve learners in which one good exemplar is better than many different exemplars. If this is the case, then using an action and a gesture to learn how to count linear units may not be helpful, particularly for children with lower prior content knowledge, or when a task is particularly difficult. In fact, in such contexts, a gesture may do nothing more than dilute the impact of an action that would have been sufficient on its own.

To determine the true efficacy of providing multiple movement-based representations of a single concept in one instructional lesson, it is also important to look for potential order effects. One possibility is that children will benefit most when they receive action-based instruction first, followed by gesture-based instruction. In many instances, children seem to learn best when they begin with more concrete representations and experiences before moving onto more symbolic or abstract ones. This is true both on a larger time scale, across ontogenetic development, and on a shorter time scale, within a single instance of conceptual development (Piaget 1953). Previous research in the domain of linear measurement shows that children with lower conceptual knowledge at pre-test learn from action-based instruction but struggle to learn from gesture, while their higher prior knowledge peers can learn from either type of instruction (Congdon et al. 2018). This cross-sectional finding suggests that as children move along the trajectory of understanding about spatial units, they understand an action-based representation of units before gesture-based representations. If it is possible to transition children from one representation to another within a single instructional setting, it could be advantageous to start with the concrete, action-based representational form before moving to the more abstract gesture.

This line of thinking has been formalized in a theory known as “concreteness fading”, which refers to the practice of transitioning from concrete representations to symbolic ones over instructional time (Bruner 1966; Goldstone and Son 2005; McNeil and Fyfe 2012; Fyfe et al. 2014). In this work, researchers show that across different domains and different age groups, introducing learners to a concrete instantiation of a concept before gradually and explicitly introducing more abstract representations of a concept is better for learning outcomes than introducing a single type of representation alone, or presenting the representations in the opposite order. More specifically, the theory outlines a three-step process for learning, with the first step being concrete (e.g., a balanced physical scale with real weights), the second step being iconic (e.g., a drawing of a scale with dots representing the “weights”), and the third step being an abstract, fully symbolic form (e.g., a balanced mathematical equation). Within the structure of such a theory, it seems plausible that gesture could serve as a more abstract type of iconic representation than action, similar to graphic or pictorial symbols, in that it represents action but in a stripped-down format without extraneous details. If gesture does serve this function, and if it can do so on the timescale of a single lesson, we should predict that action-then-gesture would be the most powerful condition for teaching children. 

In light of this longstanding, evidence-based, and intuitive sense that moving from concrete to abstract representations is likely an effective instructional strategy across many ages and contexts, is there any literature to suggest that beginning with gesture *first* and then moving to action could be an effective instructional procedure? While this possibility is not as directly supported by research, the ‘desirable difficulties’ literature provides a framework that may be relevant to understanding when and why a child might be motivated to search for the meaning of a gesture that they might otherwise dismiss as ambiguous or meaningless. The idea of ‘desirable difficulties’ is that providing children with a difficult but not impossible challenge can make them more receptive to subsequent information and, under the right circumstances, can improve learning and transfer outcomes over and above a scenario with no such challenge (e.g., Bjork 2017). In this case, an ambiguous gesture could provide the initial ‘challenge’ to a child’s cognitive system, and following that ambiguous gesture with a clear, concrete action that is explicitly linked to the gesture could immediately resolve the ambiguity in a powerful way for learners who would have otherwise struggled to understand the referent of the gesture. 

### Current Study

To begin to explore some of these questions about whether and when gesture can become a more effective tool for all learners, we turn to the domain of linear measurement. Not only has a training paradigm in this domain been successfully used to directly compare the efficacy of gesture and actions-on-objects in an instructional setting, but the researchers found that for a particular, identifiable subset of the sample, gesture was particularly ineffective at getting children to change their incorrect strategies to correct ones (Congdon et al. 2018), which allows us the variability needed to test out different ways of improving gesture’s efficacy.

Linear measurement is a great place to explore these questions from an educational perspective as well. Being able to measure length is a foundational skill in mathematics and marks the first time that children are introduced to the conventionalized idea of units in a formal school setting. Units are regular spatial intervals and can be useful in both their standardized (e.g., inches, kilometers) and non-standardized (hands, paperclips) forms. While the existence of units might seem intuitive or inevitable, units are not grounded in the obvious physical properties of objects—they are socially constructed and were quite slow to emerge over historical time (Cooperrider and Gentner 2019). For these reasons, it is perhaps not surprising that units are a conceptually difficult idea for young children to master. Educators in early primary school grades tend to take two separate approaches to teaching children about units (Smith et al. 2013). The first is to use non-standard units (e.g., paperclips) to count the length of an object or compare the length of two objects, and the second approach is to use a ruler to measure the length of the object using a read-off strategy, in which children are taught to read the number that aligns with the end of the object. While these are both important skills and can be effective ways to teach children some subcomponents of the concept of units, these two kinds of activities are rarely combined within the same lesson, which tends to mask common, persistent student misconceptions about units and how they are represented by a ruler.

Revealing these hidden misconceptions can be relatively simple. If you provide a child with a ruler and an object to measure, but you place that object at a unit marker that is not the 0-point origin of the ruler, children will produce one of two well-documented errors (Congdon et al. 2018; Lehrer et al. 1998; Solomon et al. 2015). Some children will continue to use a read-off strategy, where they simply read the number on the ruler that aligns with the rightmost edge of the object, no matter where the object starts on the ruler. Other children will revert to what is called a “hatch-mark counting strategy”, where they count the lines under the object instead of the spaces. These children produce an answer that is consistently one more than the correct answer. These errors, hatch-mark and read-off errors, are not occasional mistakes. They are robust and consistent. In an international assessment of math performance, a report of released items shows that only 29% of fourth graders (and only 20% of fourth graders in the United States) can correctly answer a shifted-object measurement question (TIMSS 2011). Indeed, the most recent iteration of this global assessment identified measurement as one of the most persistently problematic subdomains of mathematics education through at least eighth grade (TIMSS 2019), when basic misconceptions about linear measurement units are likely to persist and morph into other forms such as misconceptions about perimeter, area, volume, and angle measure.

The good news is that even brief exposure to shifted-object problems can get children to disrupt their erroneous strategies (Kwon et al. 2011) and adopt new, correct strategies (Congdon et al. 2018). In the 2018 training study by Congdon and colleagues, children were either given shifted-object training or unshifted-object training with either a small plastic unit chip aligned with the ruler units (action condition) or a thumb-and-forefinger pinching gesture aligned with the ruler units (gesture condition). Using unshifted objects during training was completely ineffective for all learners, regardless of whether they were in the action or gesture version of the condition. However, in the shifted-object training conditions, there were dramatic improvements in children’s performance. Specifically, children who began the study by counting hatch-marks showed a big increase in performance after training with either actions or gestures. The children who began the study using a read-off strategy learned well from the action, but struggled to learn from the iconic gesture. These findings are nicely supported by rigorous research on the educational trajectory of learning measurement and length concepts (e.g., Barrett et al. 2017; Sarama et al. 2011, 2022), showing that children go through distinct conceptual stages in which they may be more or less receptive to different types of instruction depending on their competencies and knowledge of the problem at the moment of instruction. Consistent with this view, one possibility suggested by Congdon and colleagues is that in the context of action-on-object or gesture instruction, it is possible that the children using the read-off strategy simply may not have understood what the gesture meant—what it was intended to represent.

In the current study, we ask whether providing children with the action-based instruction and the gesture-based instruction in a single training session might improve learning outcomes by helping children in the read-off group (previously impervious to gesture training) understand the referent of the gesture. In this four-condition design, one group of children received instruction with only gesture [G], one group received only action [A], one received action followed by gesture [AG], and one received gesture followed by action [GA]. 

## 2. Methods

### 2.1. Participants

We tested 117 1st grade students (68 females; 49 males; mean age at test: 6.97 years, SD = 0.37 years) at several Chicago area schools. Children in this sample were racially and ethnically diverse—2% of children identified as Native American; 18% as Asian; 8% as Black; 55% White; and 15% as “Multiple Identities”. In addition, 21% of the sample identified as Hispanic. Children were also from a broad range of socio-economic backgrounds. Based on a categorical income questionnaire, children in the current study reported ranges from 1 (lowest possible score, <$15,000 annual income) to 6 (highest possible score, >$100,000 annual income), though the average score reported was quite high overall (5.38 out of 6, SD = 1.34). SES was a non-significant predictor of learning in all models and thus is not included in the final analyses. Children whose parents signed a consent form participated in two one-on-one sessions one week apart in a quiet area of their school (Session I and Session II).

### 2.2. Procedure

**Figure 1:**
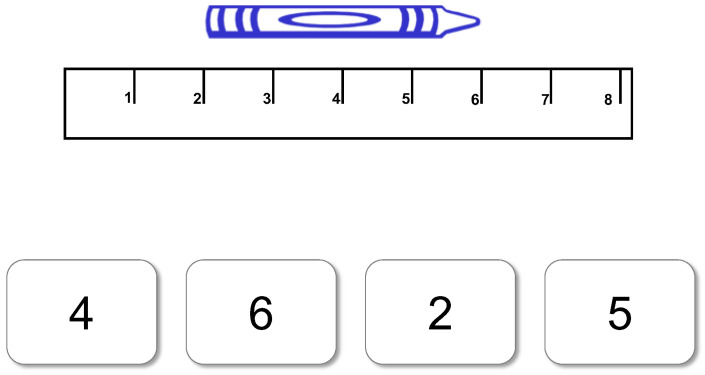
Sample multiple-choice shifted measurement test item.

Session I. To assess pre-test strategy, children were given a 14-question multiple-choice test (see Figure 1 for sample item). The first four test items were images of a crayon that was aligned with the “0” point on the ruler (“unshifted problems”). In the 10 subsequent test items, the crayons were shifted to different points on the ruler (“shifted problems”). All crayons started and ended as a whole unit. The four answer choices below the test item always reflected the correct answer: a read-off strategy answer, a hatch-mark strategy answer, and a fourth random choice that did not match any of the other three strategy-related options. This multiple-choice test was re-administered immediately after training and is the main dependent variable of interest.

Based on their responses to the pretest-shifted crayon items, children were categorized into a particular strategy group. In other words, if 6 or more of the 10 shifted-object items were answered in a way that was consistent with a single strategy, children were labeled as belonging to that pre-test strategy group. This criterion is based on the probability values of the binomial distribution: on a task with 4 possible options, answering 6 out of 10 using a particular strategy means that the child is using that strategy more often than would be predicted by chance or random guessing (*p* < .01). By this metric, we ended up with 12 children in the ‘correct’ group (N = 9 males); 46 children in the ‘hatch-mark counting’ group’ (N = 19 males); 58 in the ‘read-off’ group (N = 20 males); and 1 child (N = 1 male) whose dominant strategy was ‘random’ and did not meet criteria for inclusion in one of the other groups. Children who were in the ‘correct’ group were excluded from further analyses given that we were interested in the impact of training on performance, and these children were already at ceiling in terms of their performance. The one child in the ‘random’ group was excluded due to sample size. Two children were excluded for missing data on the follow-up session; one child was excluded due to a language barrier, and one child was excluded due to experimenter error during training. Thus, the sample represented in the final analysis consisted of exactly 100 children (n = 25 per condition), which is consistent with sample sizes in previously published work using this training paradigm (see Congdon et al. 2018).

**Figure 2:**
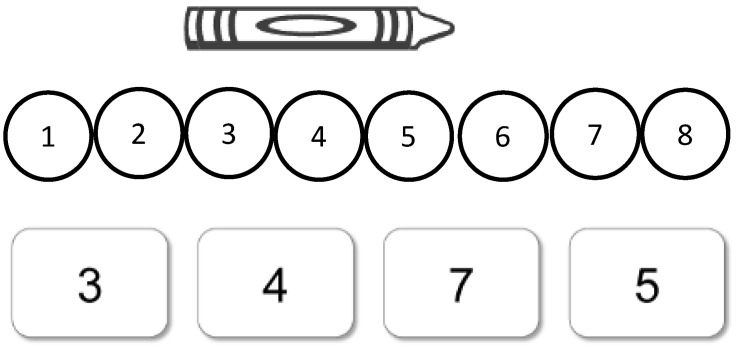
Sample item from the Numbered Circles task.

Immediately after completing the multiple-choice crayon task (pre-test), children in Session I received a set of three tasks that were intended to assess baseline knowledge on other unit-based tasks. The first task was to create an image of a crayon with numbered circles below it instead of a ruler. This task was intended to test what children would do in a slightly different type of measurement problem in a situation where there are no hatch marks to count (Figure 2). The second task involved asking children how many units long an object was, and then giving them an array of laminated unit chips to find the answer. The unit chips were either 0.75 inches or 1.5 inches long, and half of each length of unit was pink and half was yellow. The purpose of this task was to see whether participants would spontaneously select equal-sized units, or whether they would be distracted by a feature that is irrelevant to the task (the color of the units). To ensure that students were forced to contend with both of these dimensions, there were not enough chips provided of any single length or color to be able to fully measure the object. In other words, children might start with a short yellow unit, but upon running out, would need to select a long yellow unit (color match) or a short pink unit (length match). 

**Figure 3:**
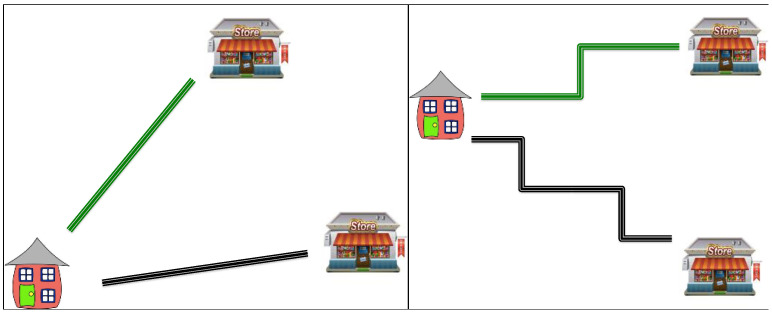
Sample Items from the “Going to the Store” task. The left panel shows an ‘easy trial’. When printed to scale, the paths differ in length by exactly 0.25 inches. The right panel shows a ‘hard trial’ where the total length of the paths differs by exactly 1.0 inches when printed to scale.

The third task was called “Going to the Store” and involved reading a short scenario to children in which a fictional character wants to take the shortest possible path to one of two stores. Participants were told to select the closer store by determining which was the shorter path, and were told to “Use the ruler if you think it will help.” Each participant was given two trials at pre-test and two trials at follow-up: the ‘easy trials’ had two straight paths, and the ‘hard trials’ had two zigzag paths (Figure 3). The goal of this task was to see whether children would spontaneously and correctly use a ruler as a tool to help them solve a challenging measurement task that involves length comparison. 

**Figure 4:**
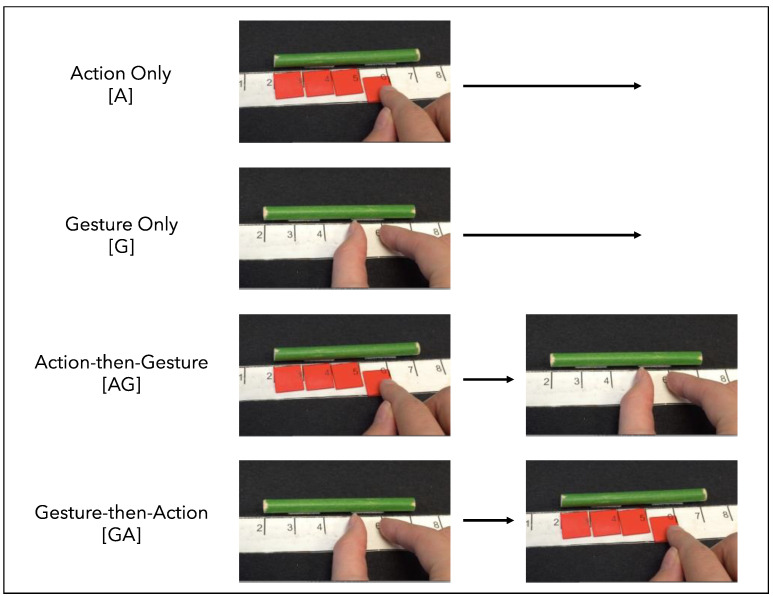
Still images of the four training conditions. The Action training conditions used small, transparent unit chips, and the Gesture training conditions used a thumb-and-forefinger ‘unit’ gesture.

After completing the baseline transfer tasks, children were randomly assigned to one of four between-subjects training conditions, which were modeled after previously published work (Congdon et al. 2018). Given the prior key finding that starting strategy is an important predictor of learning outcomes on this task, assignment to condition was intentionally counter-balanced by children’s dominant initial measurement strategy. The four training conditions were: Action only (N = 25; N = 14 read-off); Gesture only (N = 25; N = 13 read-off); Action-then-Gesture (N = 25; N = 14 read-off) and Gesture-then-Action (N = 25; N = 14 read-off). The Action training taught children to use small, transparent plastic unit chips aligned on top of the units of a ruler to measure the length of a stick. The Gesture training taught children how to make a thumb-and-forefinger “pinching” gesture to count each unit on the ruler (Figure 4).

Based on the previously published work with this paradigm showing that children do not change their incorrect strategies when they are trained on objects that are aligned at the 0-point on the ruler, training in all four conditions was performed with items that were placed away from the 0-point by the experimenter at the beginning of each trial. Before the first training trial, the experimenter introduced the materials—a set of colorful wooden sticks of different lengths, a 9-unit paper ruler, and the movement that corresponded to that child’s training condition (Action or Gesture). Then, the experimenter began the first training trial by placing one of the wooden sticks above the ruler with the leftmost edge of the stick at the 2-unit mark. The child was asked to generate a guess for how long the stick was, and then, without providing any feedback, the experimenter said, “Let us check with our unit-counter(s)”, and proceeded to count the units aloud while demonstrating the movement. Next, the child was told, “It’s your turn. Can you use the unit-counter(s) just like I did to show me how long the stick is?” After watching the child perform the movements, the experimenter said, “So, how many units long is this stick? [Wait for child reply]. Okay, let us check one more time. Count with me while I use the unit-counter(s) to double-check. [Count units]. See? The stick is X units long.” After this somewhat lengthy first trial, the remaining 7 training trials were somewhat simplified. The child was asked to generate a guess first, then asked to “check” once with their unit-counter(s), then the child and experimenter counted together while the experimenter performed the movements. The script was identical for the gesture and action conditions, as the term “unit counter” was used to describe both the plastic unit chips and the thumb-and-forefinger gesture. 

If the child performed the movements incorrectly at any point during training, they were offered a gentle correction: “Watch my hand closely while we count the units and next time, try to do what I do”. All children received a total of 8 training trials. For the training conditions with two different types of movement instruction, action and gesture, children received 4 training trials of one type and then 4 of the other, for a total of 8. During the transition from one movement type to the other, the experimenter stated, “Now we are going to play the same game, but with a different kind of unit”, and then introduced the child to the new movement type. Following training, children received a second version of the multiple-choice crayon measurement task (Posttest).

**Figure 5:**
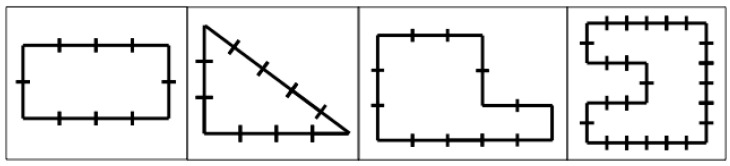
Sample items from the Perimeter Measurement task. Children were asked, “How many units would it take to go all the way around the outside edge of this shape?” This task was only administered at the second session.

Session II. Approximately one week after the second session (mean delay = 7.05 days, SD = 0.48 days), each participant received a third version of the multiple-choice crayon task (Follow-Up), followed by two brief generalization tasks that were aimed at characterizing each child’s ability to transfer his or her understanding of the concept of a “unit” to a novel context. In one generalization task, children were asked to measure three real-world objects (e.g., a toy car) with a “broken” ruler, which started with a jagged edge at the 2.5 or 3.5-unit mark. For the second generalization task, we asked children to find the perimeter of 4 different figures of varying difficulty (Figure 5). In addition, to assess growth across the training session, each child was again asked to do the numbered circles crayon measurement task, the color/size unit measuring task, and the “Going to the Store” task. 

## 3. Results

As expected, performance on the four unshifted items on the multiple-choice crayon test was nearly at ceiling already at the pre-test and remained high at all three time points (*M* = 3.93, *SD* = 0.52 at pre-test; *M* = 3.83, *SD* = 0.81 at immediate posttest; *M* = 3.68, *SD* = 1.09 at the 1-week follow-up). As such, we only carried out formal analyses on children’s performance on the ten more challenging shifted-item questions (at pretest: *M* = 0.40 out of 10.00, *SD* = 1.96). 

On the main outcome of interest, the crayon and ruler task, the data were non-normally distributed (children either got most of the 10 problems right or most of the 10 problems wrong). For this reason, the data were fit with a mixed effects binomial logistic regression model that predicted correct performance on each individual shifted-object test item. In all models, we included a random effect of participant to account for the fact that multiple data points came from the same participant. All analyses were performed using R (R Core Team 2021). 

**Figure 6:**
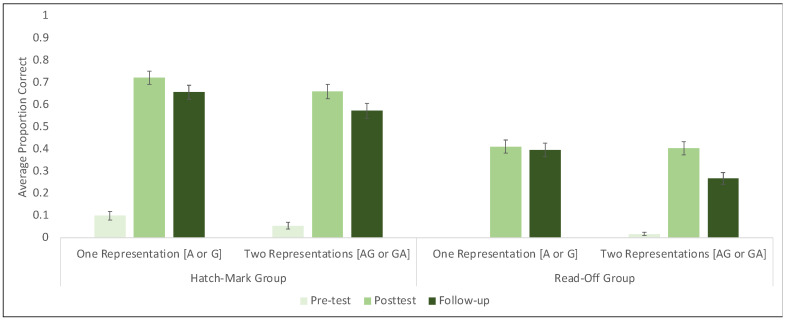
Proportion of trials answered correctly by session, strategy, and number of representations. Bars represent +/− 1 SE of the mean.

To begin, we first looked at whether there was a main effect of the number of representations (one or two) on learning outcomes. We ran two binomial regression models with trial accuracy on either the post-test session or the follow-up sessions as the two main outcome measures. Each model included the number of representations used during training (one or two) as the main fixed effect of interest, with random effects of both participant and pre-test score. Results showed that the number of representations used during training (one or two) was not a statistically significant predictor of improvement on the main outcome measure at posttest (β = 0.26, SE = 1.7, *Z* = 0.15, *p =* 0.88) or one week later at the follow-up session (β = 2.25, SE = 1.69, *Z* = 1.33, *p =* 0.18). In other words, we find no evidence that providing children with two different but complimentary representations of units during training worked any better or worse than using only one representation. This analysis remained the same even after accounting for differences based on the starting strategy (Figure 6). 

### 3.1. The Role of Starting Strategy

As is evident in Figure 6, children who began the study by using the read-off strategy learned and retained less from instruction overall than children who began the session counting hatch-marks. Indeed, previous work using a very similar training paradigm has shown that the efficacy of instruction can vary dramatically depending on children’s prior conceptual knowledge about measurement as indexed by their dominant starting strategy (Congdon et al. 2018). Specifically, those researchers found that children who began the study by counting hatch-marks improved similarly from both action and gesture instruction, but children who began by using the read-off strategy improved significantly more from the action training than from the gesture training. In other words, the authors reported a significant condition by starting strategy interaction. To account for the potential role of starting strategy in our data and specifically to test whether the effect of training condition in the current study interacts with children’s dominant pre-test strategy, we built a model with all four training conditions, pre-test strategy, and the interaction between condition and pre-test strategy as the fixed effects of interest and trial accuracy at post-test as the binary outcome measure. The model also included random effects of participant and pre-test score. 

The results of this analysis show that the difference in post-test scores between the hatch-mark group and the read-off group is most pronounced in the gesture condition (β = −12.98, SE = 5.29, *z* = −2.45, *p =* 0.014). However, none of the other achievement gaps between read-off and hatch-mark children were statistically significantly different from one another, and an analysis of variance of the model shows only a marginal overall interaction between condition and starting strategy at posttest (Χ^2^ = 7.14, *p* = .07) but a large main effect of starting strategy (Χ^2^ = 12.09, *p* = .0005). In addition, a similar analysis at the follow-up session reveals a powerful main effect of starting strategy (Χ^2^ = 10.80, *p* = .001), but no statistical evidence of an interaction between starting strategy and condition (Χ^2^ = 2.53, *p* = .47). 

### 3.2. Main Analysis

**Figure 7:**
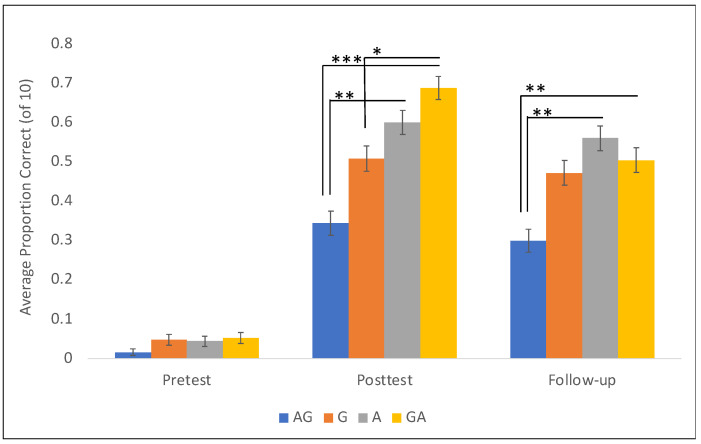
Raw means of performance at each testing session by condition. Please note that while raw means of the proportion of trials answered correctly and the standard errors of those means are plotted here for ease of presentation and interpretation, any significance markers on the post-test and follow-up data derive directly from the output of the binomial regression models, which treat the outcome measure as binary (success or failure on a given trial) and control for participant identity, starting strategy, and the small but predictive role of pre-test score. Bars represent +/− 1 SE of the mean. * *p* < .05, ** *p* < .01, *** *p* < .001.

Based on this analysis, we opted not to diminish the sample size of the study by breaking the analysis into two groups based on the starting strategy. Instead, we choose to explore the main effects of the training condition with the starting strategy as a control variable in each model (Figure 7). For our first model looking at the effect of training condition on posttest performance, there is a clear main effect of starting strategy where children who begin with the read-off strategy improve less than children who started the study with the hatch-mark strategy (β = −7.19, SE = 2.22, *z* = −3.24, *p =* 0.001). To explore the efficacy of the training conditions, we collapse across starting strategies (read-off and hatch-mark) for all analyses. We find that children in the GA condition significantly outperformed children in both the G (β = 5.65, SE = 2.85, *z* = 1.98, *p =* 0.047) and AG (β = 8.59, SE = 2.31, *z* = 3.71, *p =* 0.0002) conditions. In addition, those in the A condition outperformed those in the AG training condition (β = 6.42, SE = 2.38, *z* = 2.70, *p =* 0.007). There was no significant difference between the performance of children in the GA and A conditions. 

At follow-up, some of the effects reported at posttest had faded slightly. Children in the GA condition continued to outperform those in the AG condition (β = 5.86, SE = 2.34, *z* = 2.5, *p =* 0.01), but their performance did not statistically differ from those in the G condition (β = 2.79, SE = 2.10, *z* = 1.33, *p =* 0.18). Children in the A condition continued to outperform those in the AG condition (β = 5.36, SE = 2.24, *z* = 2.39, *p =* 0.017), and starting strategy continued to be a significant predictor of scores at the follow-up session over-and-above training condition (β = 2.94, SE = 2.59, *z* = 1.14, *p =* 0.026). Again, there was no difference between the performance of the children in the GA and A conditions. 

Overall, these findings paint an emerging picture that the gesture-then-action condition is effective at promoting learning on an immediate posttest. Children in that group significantly outperform children in both the gesture condition and the action-then-gesture conditions, meaning that if you consider the three training conditions that incorporate gesture in some manner (GA, AG, and G), GA is the clearly superior way of incorporating gesture in ruler measurement instruction. However, the effects fade somewhat at the one-week check-in, such that children in the GA condition no longer statistically outperform children in the gesture alone condition. The action alone condition is also quite strong in terms of learning. It does not differ statistically from either the gesture condition or the gesture-then-action condition at either posttest or follow-up. At the bottom of the hierarchy lies the action-then-gesture condition, in which children learned and retained significantly less than two of the three other conditions. These findings leave us with an open question about whether GA and A differ in any meaningful way that would justify the addition of gestures to the already effective A training. 

### 3.3. Transfer Tasks

To explore this question, we examined children’s performance on the transfer tasks. There were three tasks that were administered at both pre-test and follow-up to assess change across the experiment (numbered circles; colorful unit chips; ‘going to the store’), and two tasks that were administered only at follow-up (perimeter and ‘broken ruler’). For each task, we ran a linear regression model that used training conditions to predict performance on the transfer task. For the three tasks that were included in both testing sessions, the main effect of condition was subsumed by an interaction term between session and condition. In all models, we control for improvement on the main task and starting strategy. We performed an analysis of variance on each model to report the overall effects of each factor on children’s performance while controlling for each of the other factors. The results are summarized in Table 1. Of the five transfer tasks, only one task shows differential performance by training condition—the Numbered Circles task. While the Broken Ruler task did correlate nicely with improvement on our main task and thus may be capturing some carry-over effects of our training, we do not find any significant differences by condition. 

**Figure 8:**
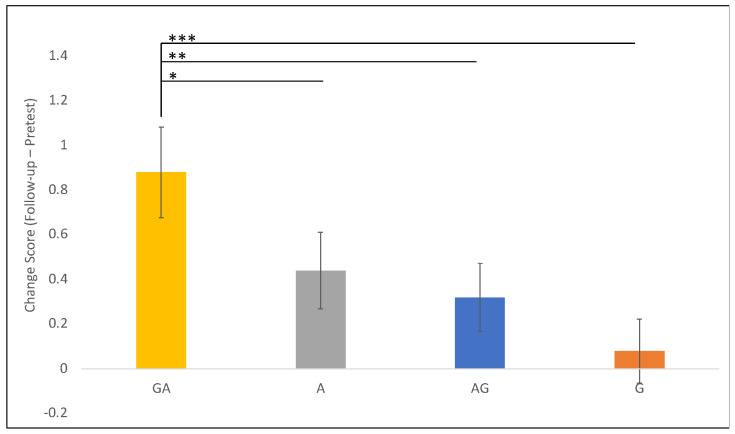
Improvement on the Numbered Circles task by condition. For ease of presentation, this figure displays raw averages of improvement from pre-test to follow-up on the numbered circles task. Significance markers indicate significant differences from the model, which looked at the interaction between session and condition and controlled for both starting strategy and improvement on the main outcome measure. The maximum possible improvement on the task was two. Bars represent +/− 1 SE of the mean. * *p* < .05, ** *p* < .01, *** *p* < .001.

Further exploration of the model by looking at performance on the Numbered Circles task shows that one training condition is much more effective than others at promoting transfer. Specifically, the improvement on the numbered circles task was statistically significantly larger in the GA group than in the G group (β = −0.4, SE = 0.10, *z* = −3.93, *p =* 0.0001), the AG group (β = −0.28, SE = 0.10, *z* = −2.75, *p =* 0.006), and remarkably, even the A group (β = −0.22, SE = 0.10, *z* = −2.16, *p =* 0.031). Despite being a relatively strong intervention for learning and intervention, children in the A only group did not significantly outperform any of the other groups on the transfer task (Figure 8). 

These findings reveal that while GA and A led to similar outcomes on the main outcome measure, preceding action with a gesture *is* shaping children’s representation of the problem in a way that allows them to think more flexibly on a related but novel task.

### 3.4. AG versus GA: Exploratory Analysis of Behavior during Training

One very clear but puzzling finding that emerges from all of these analyses is that GA and AG have extremely different impacts on children’s learning and transfer outcomes, despite the fact that they are identical in content and differ only in the order in which they present information. To look for clues as to why these two conditions, in particular, might have led to such drastically different outcomes, we decided to conduct exploratory descriptive analyses of what was happening during the training session for these two groups of children. To begin, we looked at performance during training. Recall that each child received 8 total training trials, and at the beginning of each trial, they were asked to make a guess about the length of the item before being told to double-check that answer with their assigned movement (action or gesture). Children in the AG condition would have used action for the first four trials, whereas children in the GA condition would have used gesture. So, one question was whether we could detect differences in accuracy on these trials. A trained coder watched the video recordings of each training session (n = 10 videos were excluded from this analysis due to missingness, experimenter error, or file disruption, leaving n = 90 in the sample). 

**Figure 9:**
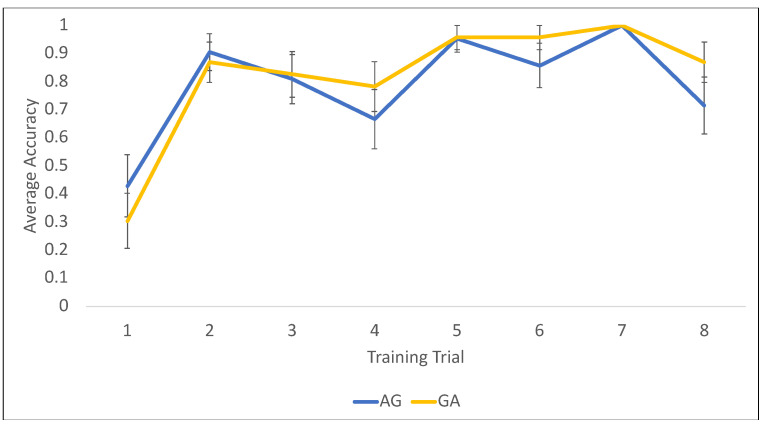
Training performance (accuracy). Bars represent +/− 1 SE of the mean.

The findings (depicted in Figure 9) do not suggest any notable differences in training accuracy as a function of condition. Across both AG and GA, children were generally likely to get the first trial incorrect, but then quickly improved and provided correct answers on the majority of the rest of the training trials. 

**Figure 10:**
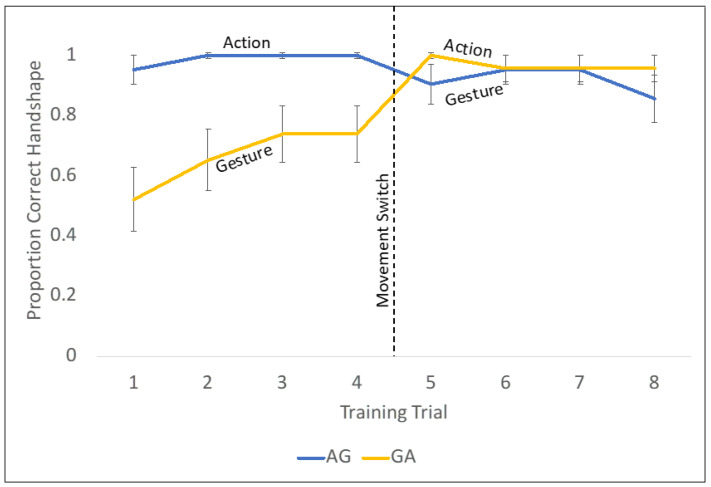
Proper form of assigned movements during training.

In addition to trial accuracy, we also examined whether children were performing the movements as intended during training. To see whether children’s ability to produce the intended movement might shed light on potential differences between the AG and GA groups during the learning process, a second trained coder looked at each training trial for each available video (n = 90) and determined whether each child was properly performing the movement on each of the training trials. A ‘proper’ movement was one that closely matched that of the experimenter and did not require corrections or additional modeling. The results of this coding are displayed in Figure 10.

Notably, this descriptive analysis reveals a stark difference in behavior between the children in the AG and the GA groups. Children in the GA group struggle to perform the iconic “pinching” gesture the experimenter is asking them to produce but then do quite well with the action movement. The reverse pattern is not observed in the AG group. The discussion section contains further speculation on how these behavior patterns during training might hint at the mechanisms of each movement type and why order is such a powerful predictor of success.

## 4. Discussion

Children vary considerably in their spatial skills (e.g., Astur et al. 1998), and this variability significantly predicts both near-term (e.g., Gunderson et al. 2012; Verdine et al. 2017) and longer-term achievement in science, technology, engineering, and math (STEM) disciplines (Mix et al. 2021), even after controlling for known third variables such as mathematics skill and verbal ability (Wai et al. 2009). In good news, a meta-analysis of interventions aimed at improving spatial thinking shows that both adults’ and children’s spatial skills are generally malleable and can be improved with practice and the proper use of tools to assist spatial thinking (Uttal et al. 2013). The current study explores the efficacy of one of these tools—gesture. Gesture can represent spatial relations and movements, and has been shown to improve learners’ performance on direct tests of spatial reasoning like mental rotation (Ping et al. 2011; Zander et al. 2016), mental transformation (Levine et al. 2018; Goldin-Meadow et al. 2012), and penetrative thinking (Atit et al. 2015), which is the skill required to imagine what the inside of an object looks like from its external features. In addition, learning a new strategy through gesture, more so than learning that same strategy through action, can lead to better transfer of novel concepts (Novack et al. 2014), perhaps because gesture can flexibly represent conceptual information that is not tied to particular objects. 

While gesture can be a powerful tool for improving performance on spatial reasoning tasks, there is remarkable variability in which learners seem to be able to take advantage of its benefits. Children’s prior knowledge of the concept being taught (Congdon et al. 2018), their own experience using gesture to communicate about a topic, and their ability to see gesture, in general, as meaningfully representational are some individual differences that may predict whether gesture is helpful to an individual child (Congdon et al. n.d.). A unifying theme in all of this work is that some children simply may not understand the intended referent of the iconic gesture they are being asked to produce and learn from, which could lead to an ambiguous representation of the target concept in the mind of the learner. In this specific case, where we use gesture to represent a spatial unit on a ruler, the flexible affordances of gesture could have some downsides—the distance between a child’s fingers could vary from unit to unit, or the “finger unit” may be especially difficult to cleanly or clearly iterate across the continuous span of the object. These properties mark a stark contrast between the gesture and the action, as the action is assisted by a moveable object with constant, predictable properties and a concrete, countable way of representing space.

The current study tests whether providing a gesture *in the context of an action* might help children understand, and thus benefit from, the unique properties of gesture, such as its reported ability to help children generalize their learning to novel contexts. We directly compare learning, retention, and generalization outcomes from four different training conditions [A, G, AG, GA] in a linear measurement learning paradigm. The first major finding is that while both the action only and gesture-then-action training conditions are quite effective at improving learning outcomes at both the posttest and the one-week follow-up session, the gesture-then-action condition dramatically outperforms all three other training conditions on our transfer task. 

Why do differences emerge between GA and A on transfer problems when there was no differential performance on the main outcome measure at either immediate or follow-up testing? This is not the first paper that shows this kind of effect, whereby gesture and action lead to similar initial learning outcomes but differences in transfer or generalization. In one study, which used either gestures or actions to teach children problem-solving algorithms for balancing a pre-algebraic equation, the authors argued that the features of gesture that differentiate it from actions-on-objects—the fact that it is not tied to a particular object, is tightly synchronized with spoken language, and can be produced with more flexible, pared-down handshapes than other kinds of action—are the very same features that help learners to flexibly apply a newly learned concept more readily to a novel context (Novack et al. 2014). This same logic in reverse has been used to explain why some mathematical manipulatives, concrete physical objects meant to instantiate a mathematical concept, may be ineffective for learners if the objects have extraneous features such as color or texture that are irrelevant to the core concept being taught (McNeil et al. 2009), or if the children see the actions they have learned as only relevant to a specific set of objects (Uttal et al. 1997). 

The differences we detect on our transfer task between children in the GA and A groups are particularly striking in the context of the current study because the training conditions are identical except for the type of representation of the unit in the first half of the training session (a thumb-and-forefinger gesture in the GA condition and a transparent plastic unit chip in the A condition). The brief presence of the gesture for the GA children is clearly doing something to strengthen or otherwise expand those children’s representations of spatial units. Importantly, gesture alone does *not* show this same effect, and previous work with this paradigm has shown that some children struggle to learn from gesture at all (Congdon et al. 2018), which means that the power of gesture is specifically boosted or unlocked when a concrete action is presented after the otherwise inaccessible gesture. 

Given that action may be helping children to disambiguate the iconic gesture and take full advantage of its powerful properties as a spatial tool, it is worth considering why the AG condition was comparatively ineffective. Indeed, our second key finding in the current study is that presenting children with more representations is not necessarily always better—the order of presentation matters. In other words, while GA was the most effective training condition overall, AG was the least effective training condition overall. Given that GA and AG are identical except for the order of the training trials, what might explain their dramatically different effects on learning, retention, and transfer? 

While we are not able to directly answer this mechanistic question in the context of the current study, we can turn to children’s behavior during the training to see whether we detect any notable differences between AG and GA. Namely, we looked at their accuracy on the 8 training trials and their ability to properly form the gesture or action movement. While there were no notable condition differences in children’s accuracy on training trials, there are significant differences between the conditions in terms of children’s ability to properly produce the movements that lead to those answers. Namely, children in the GA condition show signs of initial struggle, with only 40–70% of the children properly producing the movement during the gesture portion of the training. This initial struggle is followed immediately by a resolution in the ambiguity of the gesture when those children switch to the action instruction with unit chips (95–100% accuracy). 

Previous research has suggested that this exact pattern of learning—ambiguity or uncertainty followed by clarity—could be a particularly powerful driver of cognitive change. For example, in the study by DeCaro and Rittle-Johnson (2012), children were randomly assigned to explore and think about a novel type of math problem either before or after explicit instruction. Children were then tested on both their procedural and conceptual knowledge of the problem. While both groups showed similar improvements in their procedural ability to solve the problems, the group that explored before instruction showed significantly higher conceptual understanding (see Loehr et al. 2014 for converging evidence). Additional analyses revealed that those children also entertained more possible problem solutions, paid more attention to problem structure, and more accurately gauged their own conceptual knowledge than children in the group that received the more traditional instruct-then-practice approach. If we think of an ambiguous or opaque iconic gesture as inducing this sort of mental state—where children are noting and experiencing a gap in their knowledge—it seems reasonable that following this experience with a clear type of instruction (in this case, the action) would lead to similar procedural learning, as captured by our main outcome measure, but particularly robust conceptual learning, which we have captured with our transfer task. More generally, this phenomenon can be thought of as an extension of the ‘desirable difficulties’ theory, which states that providing children with a challenge makes them more receptive to subsequent information and improves learning and transfer outcomes over and above a scenario with no such challenge (e.g., Bjork 2017; Bjork and Bjork 2020). Gesture may very well be providing a challenge to the learners on this task, as we have evidence from the current study that it is relatively ineffective on its own and harder for children with lower conceptual knowledge (Congdon et al. 2018). In this sense, it may be helping to slow children down just enough to promote more reflection and engagement with the problem, setting the stage for deeper conceptual learning. Similar explanations may help to explain previously reported phenomena in the field of mathematics education, such as work showing that second grade children learned more about place value when instruction was provided first on symbolic numerals then on concrete manipulatives rather than the other way around (Osana et al. 2017).

### Open Questions

While these perspectives offer a possible explanation for the power of the GA condition, there remains an open question as to why the AG condition was so ineffective at getting children to revise their incorrect problem-solving strategies. Ample research suggests that beginning with a concrete action and then transitioning children to a more abstract gesture could have been a powerful instructional strategy (Piaget 1953; Goldstone and Son 2005; McNeil and Fyfe 2012; Fyfe et al. 2014; Kamina and Iyer 2009; Sarama and Clements 2009). Yet, we do not observe this pattern. One possibility is that the gesture served as a sort of interference task in this particular context—rather than building on a newly acquired conceptual foundation, maybe it confused children in the latter half of their training session to the extent that they ultimately reverted to their original strategies on the posttest. A second, related possibility is that this condition would have been more effective at a higher dose. In other words, the training session in the current study was quite short—four trials of each movement type with no break in between. Perhaps a transition from concrete movement to abstract movement would have been more effective on a longer conceptual timescale. In either case, it is worth remembering that the children in the current study are quite young—first grade—and may still be developing their comfort with learning from and producing novel iconic gestures. After all, the propensity to perceive gesture as representational increases dramatically across middle childhood (Wakefield et al. 2018), and we ought to consider how that is likely to impact children’s learning from gesture instruction. Older children or adults might reasonably show a different pattern when asked to learn from action followed by gesture.

In addition, it is worth nothing that we do not find evidence of differential transfer by training condition on any training tasks except on the numbered circles task. There are several reasons why this may have been the case. Teaching children in a manner that promotes flexible generalization is certainly the goal of education, but it is incredibly challenging to do (e.g., Catrambone and Holyoak 1989). Children may not automatically and spontaneously make analogical connections between problem contexts, and this becomes less likely as the contexts move further apart in perceptual similarity because this may test the limits of their still-developing representational systems (Barr 2010). The numbered circles task is notable in that it is perceptually similar to posttest items and differs only in that there are numbered circles rather than a ruler below the to-be-measured crayon image. In addition, the numbered circles task is designed to strongly evoke the perception and counting of discrete, rather than continuous units, with no obvious read-off answer available. These features may have allowed even the children with more fragile measurement knowledge to succeed on this particular transfer task. It is also worth noting that overall, the training in the current study is particularly brief (<5 min), and while it powerfully overturns children’s specific misconceptions about ruler measurement as evidenced by posttest scores, the more distant, more challenging, and more perceptually distinct transfer tasks may have needed more intensive conceptual training to show evidence of improvement. Future research could look at whether longer or more intensive training that includes explanations and multiple movement types might alter some of the null effects we have reported here. 

Finally, with the data from the current study, we cannot definitively pinpoint the precise role gesture is playing in the GA training condition, which led to the highest rates of transfer. If one mechanism at play is that gesture is providing children with a sort of cognitive challenge or desirable difficulty, it remains an open question as to whether that function could be served by something similarly challenging or destabilizing to children, or whether gesture—an embodied tool that has been shown to lead to particularly flexible learning—might be fulfilling this role in a unique way. 

## 5. Conclusions and Future Directions

Previous work has shown that gesture can be a very powerful spatial tool, but that it is not always accessible to all learners. To understand more about its inaccessibility, we chose to focus on an age group whose understanding of iconic gestures as representational is still in development (Wakefield et al. 2018) and a context where the specific iconic gesture has been deemed largely inaccessible to children (Congdon et al. 2018). In this context where gesture has historically not been productive, we show that providing children with gesture training followed by a more accessible action can be incredibly effective—it leads to learning and better transfer performance than either action or gesture on their own. Moreover, it is much more effective than the condition with the exact same content—the AG condition. This pattern of results is exciting as it opens up the possibility that gesture, a powerful spatial tool, need not remain inaccessible to learners who struggle to intuitively understand its referents. To the contrary, a timely resolution of the gesture’s ambiguity, in this case provided by the gesture-then-action training condition, can unlock a deeper level of conceptual understanding that is greater than the sum of its separate parts. 

Taken together, our findings underscore the need for careful consideration of when and how to incorporate gestures into a lesson. An adult modeling the use of a carefully designed representational gesture may not be enough, particularly if the child does not have the proper foundational conceptual knowledge upon which to map the gesture. Instead, care must be taken to ensure that the child is provided with the context needed to link the abstract representation to the target concept. Here, we use action to solidify this link, but other approaches ought to be considered. For example, it is not clear from this work whether allowing children time on their own to explore a difficult linear measurement problem prior to formal instruction could have had a similar effect on learning and transfer after gesture instruction. Or perhaps an alternative form of the gesture, such as a simple pointing gesture, could have avoided some of the ambiguities associated with the more complex iconic pinching gesture. As noted above, the intervention used here was very brief; further work could explore whether repeated exposure to a gesture might lead a child to insight on a longer timescale and to generalize more broadly than was captured in the current study. These questions, among others, would be fruitful directions for future research and would help to further clarify the contexts under which gesture can be a powerful instructional tool. 

## Figures and Tables

**Table 1:** Analysis of Transfer tasks.

Task Name	Effect of Starting Strategy (RO < HM)	Relation to Improvement on Main Task	Main Effect of Training Condition	Condition × Session Interaction
Numbered Circles	*F* = 318.6, *p* < .001 ***	*F* = 0.65, *p* = 0.42, *n.s.*	NA	*F* = 5.41, *p* = 0.001 ***
Going to the Store	*F* = 5.65, *p* = .02 *	*F* = 0.23, *p* = 0.63, *n.s.*	NA	*F* = 1.59, *p* = 0.19, *n.s.*
Colorful Unit Chips	*F* = 12.82, *p* < .001 ***	*F* = 0.07, *p* = 0.79, *n.s.*	NA	*F* = .98, *p* = 0.40, *n.s.*
Perimeter	*F* = 3.34, *p* = 0.07, *n.s.*	*F* = 2.93, *p* = 0.09, *n.s.*	*F* = 0.25, *p* = 0.86, *n.s.*	NA
Broken Ruler	*F* = 11.51, *p* < .001 ***	*F* = 18.92, *p* < .001 ***	*F* = 1.05, *p* = 0.37, *n.s.*	NA

* *p <* .05, *** *p* < .001, *n.s.* = not statistically significant, NA = no relevant analysis.

## Data Availability

The data for this study is available upon reasonable request.
